# Long-term effects of a community-based positive youth development program for Black youth: health, education, and financial well-being in adulthood

**DOI:** 10.1186/s12889-022-13016-z

**Published:** 2022-03-26

**Authors:** Karen Sheehan, Punreet K. Bhatti, Sana Yousuf, William Rosenow, Douglas R. Roehler, Corey Hazekamp, Han-Wei Wu, Rachel Orbuch, Tami Bartell, Kyran Quinlan, Joseph DiCara

**Affiliations:** 1 Ann & Robert H. Lurie Children’s Hospital of Chicago, 225 E. Chicago Box 33, IL 60611-2991 Chicago, USA; 2 Department of Pediatrics, Rush University, IL Chicago, USA; 3 Lincoln Medical and Mental Health Center, NY Bronx, USA; 4 Department of Pediatrics, University of Illinois at Chicago, IL Chicago, USA; 5 Northwestern University, Feinberg School of Medicine, IL Chicago, USA

**Keywords:** Childhood poverty, Child adversity, Positive youth development, Health outcomes, Educational attainment, Black youth

## Abstract

**Background:**

Childhood poverty is known to be associated with poor health. For youth living in extreme poverty, community-based programs focused on youth development are one strategy to improve health and well-being outcomes. However, very few evaluations of the long-term effectiveness of youth development programs have been conducted.

The aim of this study was to assess the long-term effectiveness of a positive youth development program (PYD), serving a segregated housing project with a history of community violence, to improve the health, education, and financial well-being of its alumni.

**Methods:**

A quasi-experimental causal comparative study design was used to study the effectiveness of the Cabrini-Green Youth Program (CGYP). CGYP alumni (mean: 16.8 +/- 7.4 years after program participation) were surveyed. For comparison, participants from the same housing project who were eligible to participate in the CGYP but did not, were identified.

**Results:**

In total, 246/417 (59%) eligible alumni were located. 221 alumni were available to be interviewed; 191/221 (86%) completed the interview survey along with 143 in the comparison group. Both groups self-identified as being Black, African American, and of Other race. Alumni were younger (34.6 vs. 38.1 years, *p* < .001), less likely to be female (62% vs. 74%, *p* =.03), and more likely to have been abused as a child (26% vs. 11%, *p* = .001). The majority in both groups reported to be in good to excellent health (83% of alumni vs. 74% of comparison group). After adjusting for comparison group differences, alumni were more likely to have completed college, 24% vs. 12% (adjusted odds ratio (aOR) 2.47, 95% CI, 1.25–4.86), and to end up with some money at the end of the month, 35% vs. 19% (aOR 2.16, 95% CI, 1.17, 3.97).

**Conclusions:**

Participation in a PYD program starting at a young age may be associated with reduced poverty in adulthood, possibly aided by higher educational attainment and resultant increased income. PYD may be an effective strategy to supplement evidenced-based poverty reducing policies. This study of a voluntary, community-based PYD program is unique in its up to 33-year follow-up and an outcome assessment that measures more than knowledge change.

## Introduction

Child poverty has been associated with poor health throughout the lifespan [1, 2]. Poverty is linked with greater likelihood of food insecurity, chronic illness, and decreased life expectancy [3–6]. Poverty also harms youth by diminishing brain growth and development [7–12] often leading to poor academic achievement and limited language development [13]. Youth who grow up in poverty lag educationally behind their wealthier peers [14, 15]. Higher household income and neighborhood socioeconomic status have been linked with greater school readiness, [16, 17] while lack of school readiness predicts later cognitive problems [18–20].

It should be noted at this point that in this paper and for this study, the terms Black and African American are used interchangeably to reflect the self-identified race that program participants identified with most. Latinx is used to denote all people of Hispanic, Latino, or Spanish origin regardless of race. The authors acknowledge that race is a social construct, which is often used to describe visible differences between people but is also used as a means of oppression that largely stems from racism [21, 22], which has devastating impacts on the health and prosperity of Black, African American and Latinx youth.

Over the last two decades, the poverty rates for non-Latinx white and Asian youth (hereafter, white) have ranged from 10-15%, while rates for non-Latinx Black (hereafter, Black and African American) and Latinx (which includes all people of Hispanic, Latino, or Spanish origin regardless of race) youth have ranged from 25% up to 50% or even higher [23]. Families of color not only experience higher rates of poverty, but earlier, more extreme and more long-lasting and intergenerational poverty [24–27].

Youth who are raised in poor households and communities are at a greater likelihood of living in poverty as adults [28, 29]. Data shows that the longer a child lives in poverty the more potential there is for them to experience negative long-term health effects [30–33]; more specifically, Fass et al. reported that an African American child who spent half or more of their childhood in poverty had more than a 40 percent chance of living in poverty at age 30, while white youth had a 25 percent chance [26]; their analyses did not include data for other races and ethnicities.

Racial, residential, economic and social segregation further concentrate the effects of poverty and deprivation [34]. Most Black youth grow up in racially segregated low-income neighborhoods [35–38], which further prevents equal access to quality education and employment opportunities [39–43]. Nationwide, close to a third of African American youth born between 1985 and 2000 were raised in segregated, high-poverty neighborhoods compared with just 1 percent of white youth [33, 34]. In 2013, 81% of poor Black youth attended segregated, high-poverty schools, compared with 54 percent of poor white youth [41, 44]. According to 2019 census data, only 16.8% of adults aged 25 or older living in poverty had college degrees, and almost 25% of adults aged 25 or older living in poverty did not graduate from high school. Another 36% had a high school degree but never attended college [45].

The answer most people give for getting youth out of poverty is policy change. However, while government assistance programs and policies to help families move out of poverty have been effective, what is not widely acknowledged is that for the poorest people the current policies, even if they are effective, are insufficient. For instance, in 2019 the Temporary Assistance for Needy Families (TANF), a welfare program, served about 2 million people although that was just 5% of the nearly 40 million people living in poverty. However, that same year only 1.6 million youth received TANF benefits, which translates to only 15% of the 12 million youth living in poverty [46]. Without policy reform, inequities will continue to negatively impact marginalized communities and youth development [47–49]. Due to these historic failures, practitioners are forced to look outside of the policy arena for hope. Positive youth development programs can offer a way for youth from historically marginalized racial and ethnic groups who are living in poverty to gain access to educational and social resources and support that they may otherwise face barriers to accessing, to improve educational achievement and economic stability.

### Overview of positive youth development

Based on the definition of the Interagency Working Group on Youth Programs, Positive Youth Development (PYD) is “an intentional, prosocial approach that engages youth within their communities, schools, organizations, peer groups, and families in a manner that is productive and constructive; recognizes, utilizes, and enhances young people’s strengths; and promotes positive outcomes for young people by providing opportunities, fostering positive relationships, and furnishing the support needed to build on their strengths.” [50].

PYD has its origins in the field of prevention. Prior to the 1990s, intervention programs for youth were primarily focused on preventing problem behaviors before they surfaced, such as teen pregnancy, substance abuse, and juvenile delinquency. In contrast to this traditional approach, which primarily focuses on deficit perspectives about young people, the PYD perspective, which emerged in the early 1990s, applies an asset-based approach that emphasizes “positive development” by focusing on developing assets that enable youth to thrive [51, 52].

As defined, PYD interventions should generally aim to provide youth with education and life skills, opportunities to engage in diverse activities and settings, and positive and sustained relationships with competent caring adults. In the setting of a PYD program, mentoring offers a flexible and adaptive PYD strategy to help youth attain a range of education and life skills. Sustained, supportive and emotionally expressive relationships with non-parental adults have been significantly associated with quality of life since these relationships can impact a range of domains including education, connections to jobs and income as well as physical, mental, and emotional health [53–55]. Social connectedness during adolescence has also been associated with positive health outcomes as adults [56] and youth themselves cite social connection as being the most meaningful component of a PYD program [57].

Several models and theories to guide and operationalize PYD programs have been suggested and debated. The 5 Cs model defines the competencies that youth need to attain positive development: Competence, Confidence, Connection, Character, and Caring and others have extended this model to 7Cs by including Coping and Control [58–64]. Other newer and emerging models present frameworks for considering the roles of restorative justice, critical consciousness, and historical racism [65–67].

### Evaluation of PYD programs

PYD has been associated with positive outcomes [68–70]. A review by Catalano et al. identified 77 PYD programs of which 25 were identified as effective [71]. Generally, while it appears that several programs have the potential to promote PYD, few have been rigorously evaluated [71]. Furthermore, despite their promise, there are relatively few published studies on the long-term outcomes of PYD programs. Experts in the field note that this is paralleled by a similar lack of longitudinal investigations of normal development among racial and ethnic historically marginalized youth [72–74]. Among those that have investigated long-term outcomes, relatively few studies have been published since the early 2000s, and even fewer were developed to serve youth living in extreme poverty or Black youth [75, 76] or to evaluate what effective means for these youth. Overall, there are far fewer reported instances of PYD programs among Black youth [77, 78]; for example, in the often cited large 4-H study of PYD using the 5Cs approach, the first longitudinal study of PYD, only 7% of participants were Black youth [79]. Furthermore, there is sparse evidence to indicate that disseminating programs based on a PYD perspective can help to address both poverty and other adverse social conditions, making sustained and multigenerational poverty less likely [80, 81], even though there is great need.

### Aims of this study

The aim of this study was to assess the long-term effectiveness of the Cabrini-Green Youth Program (CGYP), a PYD program serving a segregated housing project with a history of community violence, to improve the health, education, and financial well-being of its alumni.

This study contributes to the field of youth development in several important ways. This study of the CGYP community-based PYD program showed positive results over 30 years later. The evaluation of a PYD program delivered for over 33 years provides a longer follow-up assessment than most if not all other published long-term evaluations of a PYD program. The findings also highlight return on investment for a program serving Black youth living in a low-income segregated community, which has largely been left out of the PYD literature. A lack of evidence of the long-term effectiveness of PYD programs often prevents continued investment in these important programs.

A unique element of the CGYP is that in addition to offering key elements of PYD – skill development, opportunities to engage in diverse activities and settings, and caring relationships with non-parental adults— it provides participants access to a medical home through the CGYP medical clinic. Based on a review of the published literature, no other published descriptions of PYD programs include access to clinical care, and no published evaluation study of a PYD program has included a clinical care component, even though physical health is essential to a child’s ability to attain positive development.

## Methods

### Study setting and program participants

This study evaluates the long-term effectiveness of a positive youth development (PYD) program that serves youth from families living in extreme poverty to improve the health, educational, and financial well-being of its participants.

The Cabrini Green Youth Program (CGYP) was founded in 1984 to improve the health and life opportunities of youth living in or near the Cabrini-Green Homes, a Chicago Housing Authority (CHA) public housing development. CGYP participants lived in Cabrini-Green until demolition of the housing development in 2011. Many alumni continued to participate in the program even after they moved from the Cabrini-Green Homes; free transportation was provided.

### Program components

CGYP began as a Saturday mentoring program delivered by volunteer Northwestern University medical and law students. During the early 1990’s, CGYP added afterschool tutoring and a medical home in addition to the original Saturday programming. CGYP served children beginning at birth through the CGYP medical clinic, and beginning around age 3 or 4 years, youth participated in age-appropriate reading, tutoring, and recreation programs to meet the needs of the whole child. By linking each of these program elements, the program provided participants with consistent social support.

**Fig. 1:**
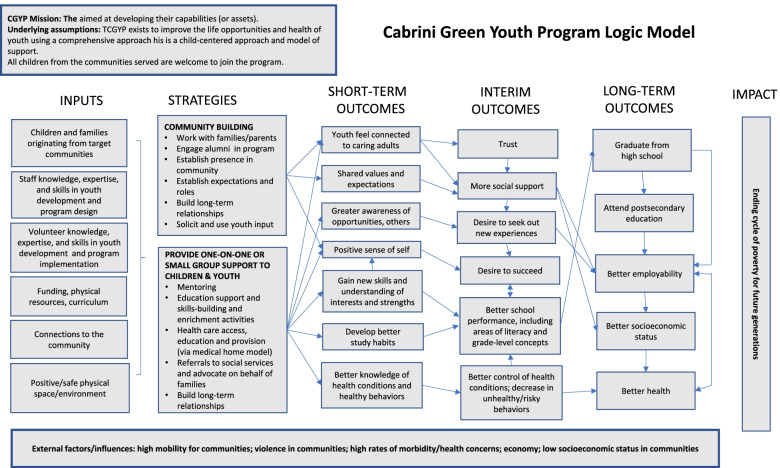
Cabrini Green Youth Program Logic Model

For example, a 6-year-old boy could attend tutoring on Monday, be seen in clinic on Tuesday, participate in a cooking class on Wednesday, and take part in a field trip on Saturday. As the participants matured, educational support included career counseling, college tours, and coaching during college or trade school attendance. Modest college or trade school scholarships up to $3,000 per year were provided. There were no eligibility requirements or cost to participate in this voluntary PYD program. A logic model for CGYP is included as Fig. 1.

### Study sample and design

Alumni were identified from the CGYP administrative data. To be included in the study, alumni had to be born between 1970 and 1995, enrolled in the program at or before age 16 years, and involved for at least two years prior to demolition of the Cabrini-Green Homes in 2011. The alumni were primarily located through referrals from other alumni or staff. In addition, the study team hired a private investigator, used social media, and employed various on-line search engines to identify participants for the study.

Using a quasi-experimental causal comparative design, a non-randomized comparison peer group was initially recruited through alumni referral. Each alum was asked to recommend someone about the same age who grew up in Cabrini-Green but did not attend CGYP. Similarly, as comparison participants were identified and interviewed, they were asked to provide names and contact information for additional comparison group members. A few comparison participants were recruited through social media groups that served former Cabrini-Green Home residents. Both former alumni and non-participants were compensated $50. Trained research assistants collected data from both groups between October 2017 and April 2019.

### Ethics approval and consent to participate

The Institutional Review Board of Ann & Robert H. Lurie Children’s Hospital of Chicago approved the study as exempt from ongoing IRB oversight and consent regulations. In accordance with federal regulations 45 CFR 46.104 and 21 CFR 56.104 and institutional review board policies, this study, for which participants were notified that their participation in the survey was voluntary, participants provided informed consent, and participants’ responses were deidentified, was determined to fall under category 2i of the categories of human subject research that are considered exempt from regulatory requirements.

### Measures

Both alumni and comparison participants completed a telephone or electronic survey of previously validated questions. Information about participant years of attendance was extracted from CGYP administrative data.

The main outcome measures for this study were self-reported health status, educational attainment, finances, and standard of living relative to their parents at a similar age. Responses were dichotomized for analyses. The first three measures were assessed using questions from the Behavioral Risk Factor Surveillance System (BRFSS) [82]. The standard of living measure was assessed with a question from the General Social Survey [83] (Table 1).

**Table 1:** Summary and source of outcome measures

Measure	Dichotomized	Source
Would you say that in general your health is:	Excellent, very good, good vs. fair, poor	Behavioral Risk Factor Surveillance System (BRFSS)
What is the highest grade or year of school you completed?	College graduate and above vs. not college graduate?	BRFSS
In general, how do your finances usually work out at the end of the month? Do you find that you usually?	End up with some money left over vs. have just enough money to make ends meet or not have enough to make ends meet?	BRFSS
Compared to your parents when they were the age you are now, do you think your own standard of living now is:	Much better or somewhat better vs. about the same, somewhat worse, much worse	General Social Survey

Demographic measures were assessed using questions from the BRFSS, Fragile Families [84], and the Centers for Disease Control and Prevention’s National Survey for Family Growth [85]. Child adversity was assessed with two measures from the Philadelphia Urban ACE Survey that were analyzed individually [86]. These items assessed direct witnessing of childhood neighborhood violence and neighborhood safety. Child physical abuse was measured with one item from the Carlson Trauma History Screen [87].

### Statistical analyses

Descriptive statistics summarized demographic and survey variables of interest stratified by program participation. P-values were calculated by a Pearson’s chi-squared test or a Fisher’s exact test in the case of small cell counts. For normally distributed continuous variables, the mean and standard deviation were reported. For continuous, but not normally distributed variables, the median and interquartile range (IQR) were reported. All statistical analysis was performed in R (version 3.5.1; R Core Team, 2018), under an alpha level of 0.05 with no adjustment for multiple hypothesis testing.

Logistic regression was used to examine associations between program participation and numerous outcomes. Simple logistic regression models were created for four major outcomes including: health status, college graduation, finances, and standard of living. Each of these outcomes were collapsed into positive and negative responses. The simple regression models included the primary exposure of program participation and the outcome of interest. In addition to the simple logistic regression models, multivariable logistic regression models were constructed to adjust for age, sex, education attainment, incarceration history, and history of physical abuse for the outcomes of health status, finances, and standard of living. For the outcome of college graduation, the adjusted model included all the variables listed above except the educational attainment variable.

Additional analyses were limited to program participants (*n* = 191). The primary exposure was length of time in the program, treated as a continuous variable. Adjusted logistic regression to control for current age and sex was conducted for the same four outcomes as in the main analysis.

## Results

**Fig. 2:**
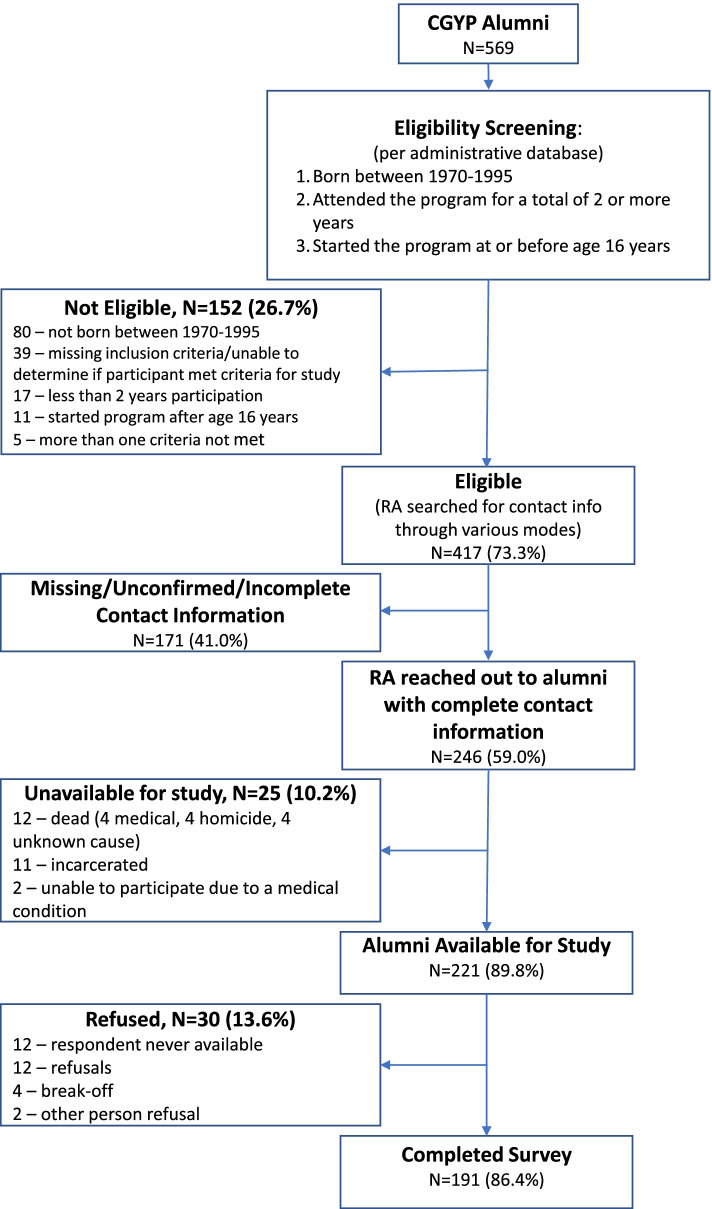
Recruitment Flowchart of CGYP Alumni

Alumni were identified from the CGYP administrative database and 417 met study eligibility (Fig. 2). Contact information was available for 246 (59%) of the eligible alumni. Twenty-five alumni were unable to participate in the study because of death, medical incapacity, or current incarceration.

In total, 191/221 (86%) of available alumni completed the survey (mean: 16.8 years, standard deviation (SD): 7.4 years, range 4-33 years). An alum (*N* = 89), a relative (*N* = 26), or a staff member or former volunteer (*N* = 32) provided contact information for 77% (*N* = 147) of the alumni who completed the survey. The remaining alumni who completed a survey were located by the following methods: private investigator (*N* = 11), social media (*N* = 12), search engines (*N* = 12), and not specified (*N* = 9). The majority of the study participants (71%) completed the survey by phone; the other alumni completed the survey online. A total of 171 (41%) former participants were unable to be located despite these extensive search efforts.

**Fig. 3:**
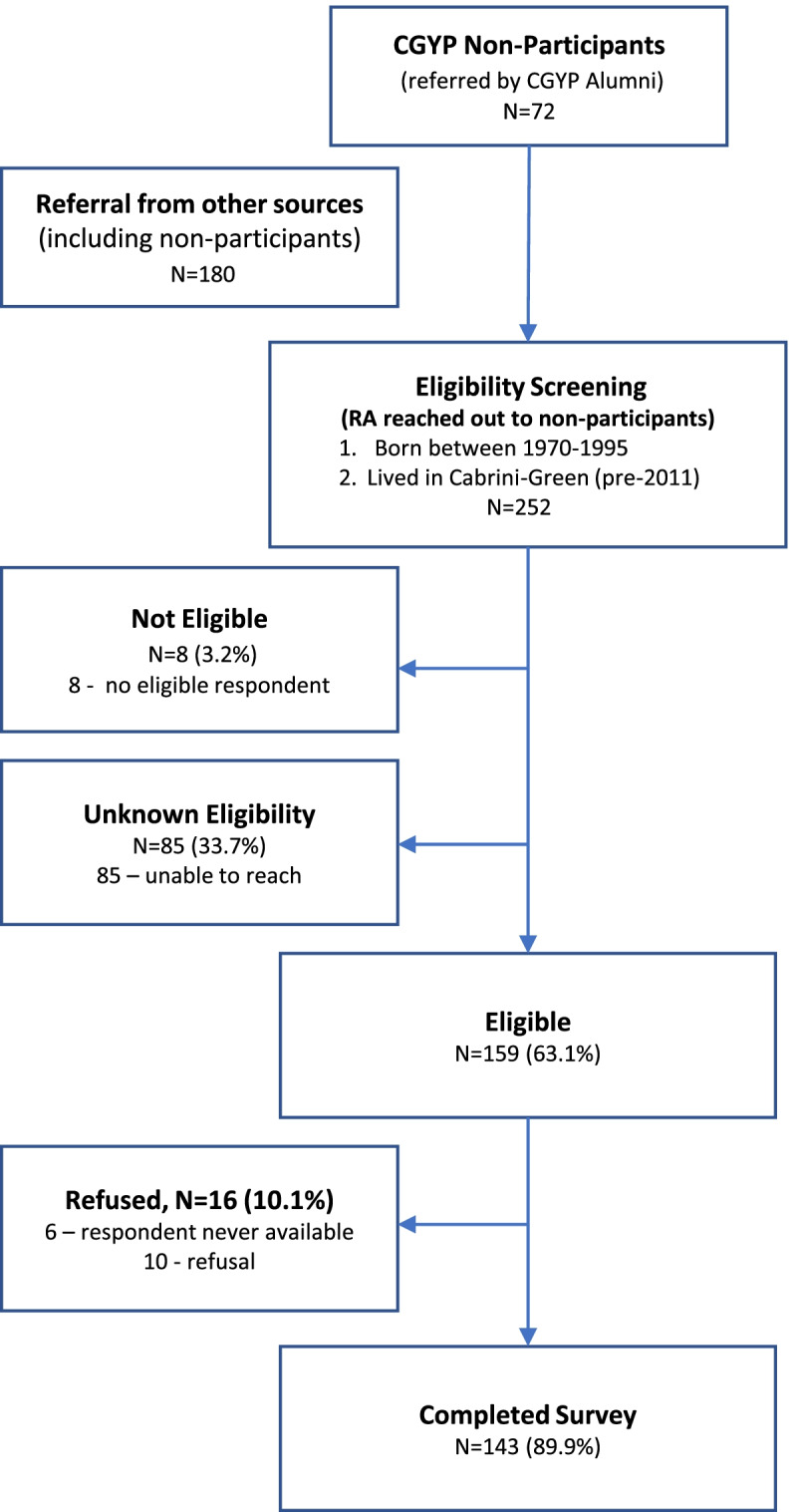
Recruitment flowchart of comparison group

A total of 143 individuals were recruited as a comparison group (Fig. 3). This comparison group was identified by alumni (17%), other comparison group participants (52%), or other/not specified (22%). Nine percent of the comparison group were recruited through social media platforms that serve former Cabrini-Green Home residents. The majority of the comparison group (92%) completed the survey by phone; the others completed the survey online.

Both groups primarily self-identified as Black and African American and reported similar experiences with neighborhood violence while growing up (Table 2). About 80% of alumni and comparison group participants report seeing or hearing, as a child, someone being beaten up, stabbed, or shot in real life. The alumni were younger than the comparison group participants (34.6 years vs. 38.1 years, *p* <.001). Among both groups, females were more likely to complete the survey with a higher percentage of comparison group females participating (74% vs. 62% former participants, *p* = .03). The alumni also reported experiencing more abuse as a child (26% vs 11%, *p* = .001). Nearly one third (28%) of alumni (*N* = 54) reported spending time in a correctional facility or on house arrest; 37 (69%) were male. In the comparison group, 19 (13%) experienced incarceration, and among those 16 (84%) were male.

**Table 2:** Demographic and early life adversity characteristics of CGYP Alumni vs Comparison Group

Characteristics	Alumni (***N*** = 191)	Comparison (***N*** = 143)	***P*** value
Current age (years), mean (SD)	34.6 (5.7)	38.1 (7.3)	<.001
Sex, N (%)			
Female	119 (62)	106 (74)	.03
Male	72 (38)	37 (26)	
Race, N (%, self-reported)			
Black	185 (97)	142 (99)	.08
Other or Missing	6 (3)	1 (1)	
Years in program, Mean (SD)	7.8 (5)	N/A	N/A
Age started in program (years), mean (SD)	8.8 (4)	N/A	N/A
Feel safe in neighborhood, N (%)			
All of the time	54 (28)	27 (19)	.07
Most of the time	54(28)	37 (26)	
Some of the time	58(30)	55 (38)	
None of the time	22(12)	24 (17)	
Missing	3 (2)	0 (0)	
How often did you see or hear someone being beaten up, stabbed, or shot in real life?, N (%)			
Many times	78 (41)	57 (40)	.39
A few times	74 (39)	58 (41)	
Once	12 (6)	13 (9)	
Never	23 (12)	15 (10)	
Missing	4 (2)	0 (0)	
Hit or kicked hard enough to injure you as a child, N (%)			
Yes	49 (26)	15 (11)	.001
No	141 (74)	128 (89)	
Missing	1 (0)	0 (0)	
Spent time in a correctional institution or house arrest, N (%)			
Yes	54 (28)	19 (13)	<.001
No	133 (70)	124 (87)	
Missing	4 (2)	0 (0)	

*Abbreviation: SD* standard deviation

Eighty-three percent of the alumni reported to be in excellent, very good, or good health compared to 74% of the comparison group, odds ratio (OR) 1.73 (95 % confidence internal (CI), 1.02-2.96) (Table 3). Alumni were more likely than comparison group participants to have completed college, 24% vs. 12%, OR 2.28 (95% CI, 1.25-4.19), and end up with some money at the end of the month, 35% vs. 19%, OR 2.36 (95% CI, 1.41-3.95). Alumni reported that their standard of living was much better or somewhat better compared to their parents at a similar age (77%) relative to the comparison group participants (68%), OR 1.89 (95% CI, 1.13-3.15).

Current age, sex, incarceration history, educational attainment, and history of being physically abused as a child differed between the alumni and the comparison group participants. When these variables were controlled for in the outcome models, college graduation and positive finances remained significantly different between the two groups (Table 3). Alumni had a 2.47 increase in odds (95% CI, 1.25-4.86) of graduating college compared to comparison group participants while controlling for current age, sex, and history of being physically abused as a child. Alumni had a 2.16 increase in odds (95% CI, 1.17-3.97) of having money left over at the end of the month while controlling for the same variables.

**Table 3:** Bivariate, Unadjusted, and Adjusted Analysis of Primary Outcome Measures Among CGYP Alumni vs Comparison Group

Outcome Measure	Alumni ***N*** = 191 N (%)	Comparison ***N*** = 143 N (%)	Unadjusted OR (95% CI)	Adjusted OR (95% CI)^**a**^
Health Status				
Excellent, very good, good	159(83)	106(74)	1.73 (1.02, 2.96)*	1.43 (0.78, 2.64)
Fair, poor	32(17)	37(26)	Reference	Reference
College Graduation				
College Graduate and above	45(24)	17(12)	2.28 (1.25, 4.19)^**^	2.47 (1.25, 4.86)*^b^
Not a College Graduate	146(76)	126(88)	Reference	Reference
Finances				
End up with some money left over each month	67(35)	2 (19)	2.36 (1.41, 3.95)^***^	2.16 (1.17, 3.97)**
Just enough money or not have enough to make ends meet	121(63)	115(80)	Reference	Reference
No response	3(2)	1(1)		
Standard of Living				
Much better or somewhat better	147(77)	98(68)	1.89 (1.13, 3.15)^*^	1.57 (0.88, 2.78)
About the same, somewhat worse, or much worse compared to parents	35(18)	44(31)	Reference	Reference
No response	9(5)	1(1)		

*Abbreviations: OR* odds ratio, *AOR* adjusted odds ratio

(*p <0.05, ^**^p< 0.01, ^***^p <.001)

^a^Multivariable logistic regression was performed to compare the difference of main outcome measures among CGYP alumni versus the comparison group. The analysis was adjusted for current age, sex, history of physical abuse as a child, education, and incarceration for the outcome measures of health status, finances, and standard of living

^b^The analysis was adjusted for current age, sex, history of physical abuse as a child, and incarceration.

Length of program participation and age at start of the program were examined to assess their effect on alumni outcomes (Table 4). For each year of program enrollment, alumni were 10% more likely to complete college (OR 1.10, 95% CI 1.02-1.17). This remained significant after controlling for current age and sex. End of month finances differed by program participation. Each year of program participation was associated with a 14% increase in odds of having extra money at the end of the month (OR 1.14, 95% CI, 1.06-1.22), after controlling for sex and age. There was no relationship between the age when a child started attending CGYP and the outcome measures (results not shown).

**Table 4:** Analysis of length of participation among CGYP Alumni (*N* = 191) and effect on primary outcomes

Measure	AOR (95% CI)
Health Status: Excellent, very good, good	
Current Age	1.03 (0.96, 1.11)
Sex (Male)	1.91 (0.80, 4.57)
Total Years in Program	1.02 (0.94, 1.11)
Educational Attainment: College Graduate or Above	
Current Age	0.97 (0.91, 1.03)
Sex (Male)	0.87 (0.42, 1.81)
Total Years in Program	1.10 (1.02, 1.17)*
Finances: End up with some money left over each month	
Current Age	1.03 (0.97, 1.09)
Sex (Male)	3.31 (1.70, 6.47)*
Total Years in Program	1.14 (1.06, 1.22)*
Standard of Living: Much Better or Somewhat Better	
Current Age	1.03 (0.96, 1.10)
Sex (Male)	0.94 (0.43, 2.05)
Total Years in Program	1.08 (0.98, 1.18)

*Abbreviation: AOR* adjusted odds ratio

* *p* < .01

## Discussion

This study identified a positive association of CGYP with the long-term educational and financial outcomes of its alumni relative to a comparison group. Alumni had improved educational and financial outcomes with each year of participation. The average age at when the alumni joined CGYP was 8.8 years old, and they were enrolled for an average of 7.8 years.

### Education and financial outcomes

Alumni were more likely to have completed college than the comparison participants, 24% vs. 12%; this is higher than the US average based on 2019 US Census data that showed only 16.8% of adults aged 25 or older living in poverty had college degrees [45]. Other studies that have examined the effect of PYD programs on academic achievement have shown mixed results [74, 88]. Measuring long-term effects of mentoring programs is challenged by the multiple diverse outcome measures used in studies (frequently limited to knowledge change) and varied length of follow up after intervention, which are rarely longer than a year or two [89–94]. This study of a voluntary, community-based PYD program is unique in its up to 33-year follow-up and an outcome assessment that measures a comprehensive set of outcomes, above and beyond knowledge change. CGYP’s results are evocative of the early education interventions that have demonstrated positive long-term impact on participants’ health and educational attainment, often decades after their pre-school experiences were complete [95–99].

In addition, CGYP alumni reported being more likely to end up with some money at the end of the month, 35% vs. 19%, which is likely due to more alumni having graduated from college. In the US, college graduates are more likely than those who are not to have a higher income [100] and it is likely that CGYP’s comprehensive educational efforts are a major contributor to this positive outcome [101].

### Health outcomes

Both groups reported moderately high levels of good to excellent health: 83% of the alumni reported to be in excellent, very good, or good health compared to 74% of the comparison group. When the model controlled for current age, sex, and history of being physically abused as a child, the differences were no longer statistically significant. Although CGYP offered access to clinical care, only about one-third of the participants were seen as patients. Since it has been assessed that only about 20 percent of a population’s health is due to access to clinical care [102, 103], it is not surprising that there was no difference in reported health outcomes for those who attended clinic and those who did not [104]. However, it is increasingly recognized that the structural determinants of health play a dominant role in health outcomes, so it may be expected that Cabrini-Green residents who grew up in a community plagued by deep poverty and segregation experienced poorer health outcomes compared to others in the state. In fact, both groups reported poorer health compared to their peers from across the state; in 2018, in Illinois, nearly 85.4% of 35–44-year-olds reported to be in excellent, very good, or good health [105].

### Exposure to community and personal violence

Both groups reported similar experiences with neighborhood violence while growing up, while alumni reported experiencing more abuse as a child. It is unclear why this difference was noted. It may be that former participants were more comfortable revealing such personal information. In any case, it does not appear that CGYP favored serving only youth without these challenges.

The findings showed that approximately 80 percent of both the alumni and the comparison group participants reported “seeing or hearing someone being beaten up, shot, or stabbed in real life.” This finding is in line with a previous study which surveyed 7 to 13-year-old youth living in Cabrini-Green Homes in the 1990s. At the time, these youth reported that, even at this young age, 42% had already seen someone shot and 37% had seen someone stabbed [106]. The reality is that the participants in this study grew up in a poor, segregated urban neighborhood with high rates of community violence [107]. Youth in economically disadvantaged areas are more often exposed to violence [108, 109], including interpersonal and community violence [110, 111], as both violence and social determinants of violence are prevalent in primarily poor urban communities [112] largely as a result of racism.

A surprising finding, especially in light of the positive effect of CGYP on alumni outcomes, is that the data reflect that alumni were more likely to report spending time in a correctional facility or on house arrest compared to non-participants. Some of this variation may be explained by the limitations of the recruitment process. Only 13% of the comparison group reported spending time in a correctional facility or on house arrest, which seems low considering that Chicago neighborhoods with similar demographics to the former Cabrini-Green Homes report that over 40% of non-Latinx Black males and 10% of non-Latinx Black females were ever in jail, prison or on probation [113]. The finding that 29% of former CGYP participants reported spending time in a correctional facility (plus the 11 alumni currently incarcerated and unable to complete the survey) parallels this similar population. It may be that comparison participants with a history of criminal justice involvement were less likely to participate.

Conversely, it is more likely that structural issues related to incarceration drive this reality, particularly for Black and African American males. Several programs in Chicago have been effective in decreasing juvenile arrests [96, 114] but incarceration is a complex issue for people of color, and particularly so for Black youth in Chicago and throughout the US, because of significant disparities in arrest, conviction, and sentencing rates, especially as compared to white youth [115]. It has been shown that youth of color are targeted by stop-and-frisk policies in their communities, and by a discriminatory school discipline and juvenile justice system that fuels a cradle-to-prison pipeline [41, 116, 117]. Until racial and structural disparities are eliminated in the criminal justice system, the impact of any program to reduce criminal justice involvement will be reduced.

### The (often) unmeasured value of social connection

Social connection with caring adults has been identified as a key tenet of PYD [101, 118, 119], and connections with non-parental adults are particularly important for youth of color, especially those living in poverty [57, 120]. Most published evaluations of PYD do not report outcomes for social connection [68–71, 80]. While this study did not measure the level of social support that CGYP participants received, findings from prior qualitative studies of CGYP [101, 119] suggest that a key element of CGYP’s effectiveness is providing youth with long-term connectedness to caring adults in a safe space.

### The importance of PYD for Black youth who experience deep poverty

Segregation itself is not an inherently place-based risk factor, but it is what can make a place a risk factor for poor health and life outcomes. Though the findings from this study for the overall effectiveness of the CGYP are limited, over 30 years of experience providing the CGYP for youth living in extreme poverty has emphasized the social and environmental deprivations that can challenge the full expression of PYD and has provided several lessons learned.

Chicago has a large gap in rates of upward mobility for Black residents compared to white residents from low-income families [121]; a likely contributor to this disparity is growing up in highly segregated and intentionally and historically underresourced neighborhoods that offer limited opportunities for its members [122, 123]. Residential segregation further concentrates the effects of poverty and deprivation – hence the need for a dedicated PYD program for youth living in the highly segregated community of Cabrini-Green. While Cabrini-Green no longer exists (and since 2000 over 80% of Chicago public housing has been demolished [124, 125]), Chicago stands as one of the most segregated cities in the US while also being one of the most diverse [126]. These facts necessitate the provision of quality out-of-school programs in low-income segregated communities with underfunded and underperforming schools, especially since the policy of tying school funding to property values ensures that schools in poor communities are underfunded [127–129].

### Policies to reduce poverty are necessary but insufficient

Despite their tremendous influence on health and well-being, poverty and deprivation are not easily treated within the clinic setting [130, 131]. However, several evidence-based policy strategies have been found to be effective at decreasing child poverty.

The National Academy of Sciences reported in *A Roadmap to Reducing Child Poverty* that the Earned Income Tax Credit (EITC), Child Tax Credit (CTC), and Supplemental Nutrition Assistance Program (SNAP) demonstrate the greatest poverty-reducing effects of current major federal assistance programs [132]. The $1.9 trillion American Rescue Plan (ARP) that was passed in March 2021 [133] included an increase in the CTC for families with low or no income from $2,000 a year per child to $3,000 per year for youth ages 6 to 17 years and $3,600 for youth under age 6. Now that eligible parents have received these monthly payments, everyone is waiting to see what impact they made for children, especially among families living in poverty, because for this CTC, unlike the earlier CTC in which poor families got a smaller benefit or nothing at all, even the poorest families were able to benefit. While the ARP was temporary, efforts are being made to make the increased CTC permanent. The Urban Institute projected that the ARP would lift 16 million people out of poverty and estimated that the poverty rate would fall 42% among non-Latinx Black families, 39% for Latinx families, and 34% for non-Latinx white families [134].

Ultimately, a key result from this long-term evaluation of the CGYP is the realization that what defines the effectiveness of a PYD intervention may not be as directly related to the program components themselves as much as to how the intervention relates to the structural reality of its participants such that – if nothing in a youth’s environment changes – even if the youth develops assets, will it matter? The limited findings and their implications suggest that the answer is yes. This is a testament not only to the impact that PYD programs like CGYP can make, but to the resilience of the youth themselves.

### Limitations

This study is not without limitations. Despite tremendous efforts to track down former participants, these attempts were unsuccessful at obtaining contact information for 40% of the alumni as many were displaced from their homes. However, based on the best available knowledge, the study population included in this study was reflective of the youth who participated in CGYP.

Over half (102/171) of the alumni who were unable to be located began the program before there were paid administrative staff, prior to 1993. The pre-1993 attendance data was handwritten and often lacked full names and complete birthdates, which made locating individuals 25 years later from a neighborhood that no longer exists very challenging. By not being able to include these missing alumni’s responses, the results may look more positive than they actually are. However, of the alumni contacted, the survey completion rate was 86%. The efforts required to collect this data were not inconsequential.

It also was a challenge to define participation [135]. An alum was defined as having a minimum of two years of CGYP participation, since in other studies short-term mentoring had negative effects [136]. However, it may have been that one year of participation would have been sufficient exposure to effect change. Unlike other studies, no difference in outcomes was observed according to what age a youth enrolled in the program, however that may have been due to insufficient power [92]. In addition, youth moved out of Cabrini-Green Homes at varying ages because of the staged approach to housing demolition; some youth continued in CGYP after moving from the neighborhood, but others did not. This led to varying length of follow-up from the end of program participation to the completion of the survey, even when compared to former alumni who were the same age. There is also the possibility that moving out of Cabrini-Green Homes was the driver of outcome improvement, although the comparison group had similar opportunity to move but had fewer positive outcomes, so this does not seem to be the primary mechanism. Overall, this study aimed to assess long-term health and financial outcomes by applying rigor to evaluate a field-based study of an intervention that was not initially conceived in a research framework, and by utilizing a variety of strategies to address confounding given the field setting. Like many other programs to promote PYD reported in the literature, CGYP was implemented without a specific plan for evaluation.

This study is also limited by potential selection bias of CGYP participants. Both alumni and non-participants experienced similar amounts of community violence during their youth, but more alumni reported more abuse as a child. On these and other demographic measures, it does not appear that CGYP enrolled only youth who were likely to do well. Any child from the Cabrini-Green area was welcome to join the program, but there may be unmeasured differences between those who joined and those who did not. Thus, alumni were likely to fare better on outcome measures than non-participants with or without the program. Without a randomized controlled trial to evaluate the CGYP intervention, the question of selection bias is not settled. Selection bias in a voluntary attendance program almost certainly exists at some level. CGYP’s impact is likely an interaction of a receptive youth to the intervention of an effective program. Furthermore, due to the observational nature of the study, while associations between CGYP and participant outcomes were identified, these do not imply causal relationships.

### Future research directions

Future studies to evaluate the long-term outcomes of PYD programs should study the timing of the initiation of PYD programming and the length of service to be able to answer questions such as: What is the minimum length of time a program should be to produce positive outcomes? Future studies also should look more closely at the role of positive non-parental adults to be able to answer questions such as: Who should serve as the adult role models—individuals who are more similar in background to the youth served, different, or both?

Recent scientific advances consistently report that exposure to adversity, particularly prolonged and sustained poverty and oppression, including intergenerational transmission, results in “toxic stress” which can affect gene expression and brain development [137, 138]. As a result, this exposure can significantly impact educational attainment and economic status. Consequently, there is an even greater need for future studies to measure diverse processes and outcomes for youth who experience toxic stress over time [139].

### Implications for the PYD model

While the PYD model has been generally supported by practitioners who develop and lead programs for a range of youth populations, PYD has faced considerable criticism for its primary emphasis on individual level change without a strong emphasis on the contexts that youth live in that can inhibit positive development, especially those most relevant to the lived experiences of Black youth [140, 141]. The literature on thriving considers four domains of ecological assets: individuals, physical and institutional resources in the social environment that provide positive opportunities for learning and recreation; collective activity or engagement between community, parents, youth, school personnel and society; and accessibility [63]. This discourse highlights the complex interplay between individual level factors, activity involvement and neighborhood assets, and that the impact of each asset depends on the context. Context clearly matters yet is not directly considered in the PYD model.

Several published papers have stressed the need for PYD to evolve, even in the early years of PYD [72, 142–146]. The particularly pointed perspective presented by Coll et al. [72] highlights concern about the lack of PYD models for conducting research that considers the diversity and strengths of racial and ethnic historically marginalized youth, and that looks beyond competencies to address and assess adaptation to adverse social contexts created by social stratification such as the effects of segregation, while introducing an integrative model that considers these factors. More recent papers have extended on this premise to include a resilience perspective [147–150] at the individual and community level [140, 151–155], though these have not always included a discussion about the impact of race or class. And as several experts in youth development have noted [140, 142–145, 148, 149, 153, 156, 157], the lack of attention to crucial aspects of the context in which youth develop particularly undermines a comprehensive understanding of the lives and development of racial and ethnic historically marginalized youth who live in poverty. This absence impedes the ability of practitioners to effectively intervene to lessen the harmful effects of challenging conditions experienced by those youth who are more likely to experience deep poverty. This is especially important since these influences often inhibit rather than facilitate positive youth development [72, 73].

In relation to the evaluation of CGYP, as noted by others the current PYD model limits the examination of the complex social, economic and political forces that impact the lives of Black youth who struggle with poverty and racism [158]. An extended model could bring greater attention to how youth navigate these issues. While newer studies authored by early developers, adapters and implementers of the PYD model have considered the role of context, future adaptations must extend descriptions of contexts to include racial discrimination and stigma, institutional racism and structural racism [159] to address racial equity [160] and challenge systems of oppression through a reimagining of what is possible through PYD programs [161].

## Conclusions

The findings from this unique long-term study of a 33-year PYD program suggest that participation in CGYP (which has expanded to and continues as the Chicago Youth Programs (CYP) [162] and is now operated by paid staff and delivered by over 600 volunteers), which provides participants with a long-term commitment to provide educational support and life skills, access to diverse opportunities and settings, and social connectedness to caring adults, is associated with reduced poverty in early adult life. The effectiveness of the CGYP model is likely facilitated through the higher educational attainment of its alumni and their resultant higher income. While this study had significant limitations, this finding is of vital consequence since it points to the potential of PYD programs like CGYP that can provide resources and additional support for those who are the most marginalized, and that have significant potential to interrupt the cycle of intergenerational poverty by increasing education and financial outcomes for youth who take part in those programs.

### Importance and relevance

For youth living in deep poverty, CGYP may be an effective model of positive youth development, particularly for those who continue to face persistent social inequities. While PYD programs have shifted toward an asset-based approach, this shift has been slower for youth of color and many ongoing efforts still primarily focus on risk-based assessments primarily among programs that include Black youth. Based on the best available knowledge, CGYP was also the first and is still the only PYD program to offer participants access to clinical care. Further studies are needed, and encouraged, to help illuminate the value of PYD for youth of color living in poverty and to reimagine what is possible, perhaps particularly for those youth who routinely experience abuse and witness violence. The hope is that articulating these findings can contribute to interdisciplinary efforts leading to the advancement and necessarily evolution of PYD for Black youth living in poverty who can benefit from them.

## Abbreviations

aOR: Adjusted odds ratio
BRFSS: Behavioral Risk Factor Surveillance System
CGYP: Cabrini Green Youth Program
CHA: Chicago Housing Authority
CI: Confidence internal
CTC: Child Tax Credit
CYP: Chicago Youth Programs
EITC: Earned Income Tax Credit
IQR: Interquartile range
OR: Odds ratio
PYD: Positive Youth Development
SD: Standard deviation
SNAP: Supplemental Nutrition Assistance Program

## Declarations

KS and JD are co-founders of the Chicago Youth Programs. They did not participate in the analysis of the data.
