# Correction to: Long-term effects of a community-based positive youth development program for Black youth: health, education, and financial well-being in adulthood

**DOI:** 10.1186/s12889-022-13177-x

**Published:** 2022-04-21

**Authors:** Karen Sheehan, Punreet K. Bhatti, Sana Yousuf, William Rosenow, Douglas R. Roehler, Corey Hazekamp, Han-Wei Wu, Rachel Orbuch, Tami Bartell, Kyran Quinlan, Joseph DiCara

**Affiliations:** 1 Ann & Robert H. Lurie Children’s Hospital of Chicago, 225 E. Chicago Box 33, Chicago, IL 60611-2991 USA; 2 Department of Pediatrics, Rush University, Chicago, IL USA; 3 Lincoln Medical and Mental Health Center, Bronx, NY USA; 4 Department of Pediatrics, University of Illinois at Chicago, Chicago, IL USA; 5 Northwestern University, Feinberg School of Medicine, Chicago, IL USA

**Correction to: BMC Public Health 22, 593 (2022)**

**https://doi.org/10.1186/s12889-022-13016-z**

The original publication of this article contained 2 errors:

1. The top left gray box was corrupted in figure 1
2. The sentence :
  - “The hope is that articulating these findings can contribute to interdisciplinary efforts leading to the advancement and **necessarily** evolution of PYD for Black youth living in poverty who can benefit from them.”
  Should have read:
  “The hope is that articulating these findings can contribute to interdisciplinary efforts leading to the advancement and **necessary** evolution of PYD for Black youth living in poverty who can benefit from them.”

These do not impact the conclusion/results of the article. The original article has been updated with the correct information.

